# Management and Mitigation of Vibriosis in Aquaculture: Nanoparticles as Promising Alternatives

**DOI:** 10.3390/ijms241612542

**Published:** 2023-08-08

**Authors:** Nuan Anong Densaad Kah Sem, Shafinaz Abd Gani, Chou Min Chong, Ikhsan Natrah, Suhaili Shamsi

**Affiliations:** 1 Department of Biochemistry, Faculty of Biotechnology and Biomolecular Sciences, Universiti Putra Malaysia, Serdang 43400, Malaysia; gs63821@student.upm.edu.my (N.A.D.K.S.); shafinaz_abgani@upm.edu.my (S.A.G.); 2 Department of Aquaculture, Faculty of Agriculture, Universiti Putra Malaysia, Serdang 43400, Malaysia; choumin@upm.edu.my (C.M.C.); natrah@upm.edu.my (I.N.)

**Keywords:** vibriosis, *Vibrio* spp., aquaculture, graphene oxide

## Abstract

Vibriosis is one of the most common diseases in marine aquaculture, caused by bacteria belonging to the genus *Vibrio*, that has been affecting many species of economically significant aquatic organisms around the world. The prevention of vibriosis in aquaculture is difficult, and the various treatments for vibriosis have their limitations. Therefore, there is an imperative need to find new alternatives. This review is based on the studies on vibriosis, specifically on the various treatments and their limitations, as well as the application of nanoparticles in aquaculture. One of the promising nanoparticles is graphene oxide (GO), which has been used in various applications, particularly in biological applications such as biosensors, drug delivery, and potential treatment for infectious diseases. GO has been shown to have anti-bacterial properties against both Gram-positive and Gram-negative bacteria, but no research has been published that emphasizes its impact on *Vibrio* spp. The review aims to explore the potential use of GO for treatment against vibriosis.

## 1. Introduction

**Figure 1:**
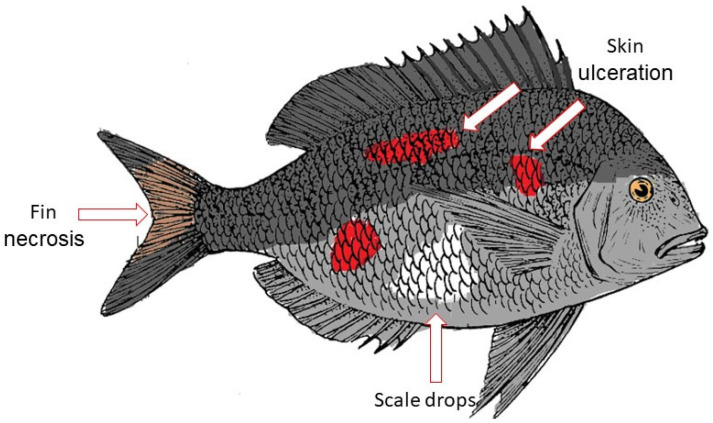
Vibriosis in fish. Vibriosis symptoms in fish, as shown by the skin ulceration, scale drops, and fin necrosis.

Vibriosis is one of the most common diseases in marine aquaculture, caused by bacteria belonging to the genus *Vibrio*. It is a dangerous epizootic disease that affects both wild and farmed marine species all over the world [1]. Diseases can have an impact on aquaculture production by destroying basic resources, diminishing the physical output or unit value of a production process, reducing its efficiency, and directly affecting human health. Vibriosis has become a challenge for the growth of the aquaculture industry [2]. Economic losses due to disease outbreaks in the aquaculture sector are expected to exceed over USD 9 billion annually, or 15% of the value of farmed fish and shellfish production [3]. In Malaysia, vibriosis has been reported in highly valuable commercial finfish species such as groupers and Asian seabass. The disease has been found to cause significant mortality in cultured groupers in floating fishpens and net-caged Asian seabass [4]. Vibriosis was reported to have caused a total loss of EUR 0.19 per kilogram of tail, or 7.06% of the overall production cost of Asian seabass per kilogram [2]. *Vibrio* species are linked to infections in fish that cause symptoms such as skin ulceration, scale drops on the stomach, and caudal fin necrosis, as shown in Figure 1 [5]. Hemorrhagic septicemia, ulcers, cholera, gastroenteritis, and skin infections are the most common symptoms of vibriosis in humans [6]. The majority of cases have been connected to the consumption of raw or undercooked seafood, as well as the consumption of polluted water.

The rapid expansion of the aquaculture industry resulted in outbreak and spread of vibriosis, which caused massive losses for aquaculture industries around the world. Aquaculture faces significant difficulty in the prevention of vibriosis [7]. Antibiotics are currently one of the most extensively utilized treatments for vibriosis in aquaculture. However, uncontrolled use of antibiotics has resulted in an increase in multidrug-resistant (MDR) bacteria, which limits the treatment of bacterial illnesses in animals as well as in humans [8]. Antibiotics have been outlawed in several nations because the residues are harmful to human health and the environment. Alternative treatments for vibriosis, such as vaccination and probiotics, have shown significant effects. However, vaccination has shown inconsistency of performance in on-site field application and it is not cost-effective due to the high labor costs that result from the repeated or frequent administration of vaccines through injection. In addition to that, animals could also be subjected to repeated animal restraint, painful injections, and sharps hazards following the injection of vaccines, which could add more trauma and additional stress for the animals [9]. On a different note, probiotics have been used as alternatives, but the major hurdle lies in the unknown concentration of probiotics that need to be administered for them to be deemed efficacious. The administration of probiotics could also lead to the addition of unnecessary bacteria to the pool of pre-existing bacteria in the animals, which could trigger bacterial poisoning that could exacerbate organ failure and death [10]. Drug resistance has become widespread among many species of pathogenic bacteria globally, resulting in the ineffectiveness of the most commonly used antibiotics in the treatment of infectious diseases, causing concern and challenges in the healthcare industry [11]. The rise of MDR bacteria has left plenty of room for scientists to discover new forms of alternatives against vibriosis, which include the application of nanotechnology.

## 2. Vibriosis

### Characteristics of Vibrio Spp.

*Vibrio* spp. are Gram-negative bacteria that cause vibriosis. The bacteria are straight or comma-shaped rods with polar flagella enclosed within a sheath [12]. Vibriosis is a water-borne infection, which means that the causative agent spreads through the water column. *Vibrio* species live in a variety of aquatic habitats, including rivers, estuaries, seas, and deep-oceanic waters. The presence of *Vibrio* bacteria can be influenced by temperature, pH, salinity, and nutrients in the aquatic environment [13]. They are thermophilic bacteria that are known to be highly adaptive and capable of surviving in seawater under adverse conditions [14]. *Vibrio* grow best in warm (>18 °C) seawater and brackish waters with high nutrient concentrations. Water with a pH of 7.5 to 8.5 is reported to be optimum for *Vibrio* spp. growth. *V. cholerae*, *V. parahaemolyticus*, and *V. vulnificus* can grow in water with a maximum salinity of 10 parts per trillion (ppt) [15]. *Vibrio* species have been discovered to be opportunistic pathogens, meaning they cause disease when the host’s immune system is suppressed or weakened due to physiological or environmental factors [16]. Pathogenicity appears to be influenced by strain characteristics, since certain strains from the same species can be extremely pathogenic while others are non-pathogenic [17].

## 3. Treatment against Vibriosis and Its Limitation

### 3.1. Antibiotics

Antibiotics are the most common treatment used in aquaculture to treat bacterial infections, and most *Vibrio* spp. are susceptible to them. Antibiotics are widely used in bath treatments or mixed with feed for therapeutic usage. Antibiotics such as oxytetracycline, tetracycline, quinolones, nitrofurans, potentiated sulfonamides, trimethoprim, sarafloxacin, flumequine, and oxolinic acid have been used to treat vibriosis [18]. The most frequently utilized antibiotics are tetracycline and quinolone antibiotics, as well as sulfonamides, which are typically potentiated with trimethoprim. El-Gohary et al. [19] reported that the mortality rate of infected Nile tilapia fish (*Oreochromis niloticus*) by *V. alginolyticus* was reduced after being treated with florfenicol (25 mg/kg), enrofloxacin (50 mg/kg), and oxytetracycline (50 mg/kg) for 7 days. In another study, orally administered 10 and 20 mg/kg of florfenicol, 25 mg/kg of oxolinic acid, and 25 mg/kg of flumequine were found to help lower cumulative mortality in lumpfish (*Cyclopterus lumpus*) infected by *V. anguillarum* [20]. Treatment with oxolinic acid combined with oxytetracycline showed positive effects in white shrimp (*Penaeus vannamei*) challenged with *V. parahaemolyticus*. The combination of antibiotics was successful, with the survival rate of shrimp significantly increasing and the muscles showing no signs of drug residues. The research revealed that 50 mg/kg oxolinic acid and 50 mg/kg oxytetracycline have more effective antibacterial effects against *Vibrio* sp. Combination treatment with antibiotics could be a new strategy in aquaculture [21].

However, antibiotic resistance has developed in pathogens due to the widespread and frequent use of antibiotics in aquaculture in the past. The emergence of resistant bacteria and resistant genes can have an impact on treatment effectiveness and public health by spreading antibiotic-resistant bacteria to consumers [22]. A large number of resistant genes have been reportedly found in bacteria and the environment. Antibiotic resistance genes can be passed down through vertical gene transfer to the next generation or exchanged with other bacteria through horizontal gene transfer [23]. The resistance genes include penicillin resistance genes *penA* and *blaTEM-1*, as well as tetracycline resistant genes *tatA*, *tatB*, *tatC*, *tatD*, *tatE*, *tatG*, *tatH*, *tatJ*, *tatY*, and *tatZ* [24]. Ampicillin, penicillin, and tetracycline resistance is the most commonly detected antibiotic resistance for *Vibrio* spp. [25].

Several studies have reported the presence of antibiotic-resistant bacteria. Grouper samples revealed the presence of *V. parahaemolyticus*, *V. alginolyticus*, *V. vulnificus*, *V. rotiferianus*, *V. campbellii*, *V. mytili*, *V. furnissii*, *V. harveyi*, *V. tubiashii*, *V. fluvialis*, and *V. diabolicus*, which were resistant to ampicillin, penicillin G, and vancomycin. *Vibrio* spp. lost their plasmid throughout the curing process, but they remained resistant to ampicillin, penicillin G, bacitracin, and vancomycin. The findings suggest that antibiotic resistance in *Vibrio* spp. could be linked to chromosomal and plasmid-borne factors [26]. A previous study reported that *V. parahaemolyticus* was highly resistant to ampicillin [27]. Similarly, You et al. [28] revealed that *Vibrio* species isolated from the west coast of Peninsular Malaysia were resistant to ampicillin. The ampicillin-resistant pattern could be attributed to the abuse of first-generation antibiotics, such as ampicillin, in the environment, resulting in reduced susceptibility and low efficacy of ampicillin in *Vibrio* infection treatment [29].

Antibiotic contamination has been shown to influence water-quality metrics and the structure of natural bacterial populations, resulting in effects on endpoints defining aquatic ecosystem functioning processes [30]. Furthermore, the frequent use of large amounts of antibiotics has the potential to cause significant levels of bioaccumulated residues in food chains, eventually causing secondary harm to non-target species [31]. Wild fish may consume antibiotic residues, which would affect the safety of aquatic products [32]. The intake of residue antibiotics in aquatic products has led to allergic reactions and toxicological issues in consumers. Human bioaccumulation of residues results in “chronic toxicity,” which damages organs. Unprotected workers in the aquaculture sector can suffer from allergies to antibiotics and problems of toxicity, as they risk being exposed to large doses of antibiotics during the mixing of antibiotics with feed and dispersal in ponds. It is widely known that exposure to several antibiotics, such as sulphonamides, can cause contact dermatitis [33].

### 3.2. Vaccination

The consequences of antibiotic abuse have demanded the development of novel techniques to combat vibriosis. Techniques including vaccinations are being investigated for the prevention and control of vibriosis in aquaculture, with promising results. A vaccine is a non-polluting biological agent that provides enhanced immune protection by inducing immunological memory that is well targeted and long lasting. The administration of vaccine can trigger immune response in the recipients’ body. The targeted antibodies can neutralize and eradicate pathogenic microbes and the toxins they create, providing superior protection against pathogenic microorganisms [34]. A study by Nor et al. [35] showed that intraperitoneal administration of marine red hybrid tilapia (*Oreochromis niloticus* × *O. mossambicus*) with dead *V. harveyi* improved the fish’s vibriosis resistance and antibody response. The study showed that vaccinated fish had a higher survival rate (87%) compared to unvaccinated fish (20%). In addition, the live attenuated vaccine was used to treat *Vibrio* infections. The live attenuated vaccine requires the use of mutation and other therapies to minimize the toxicity of the pathogen while retaining its immunogenicity. It allows the attenuated pathogen to multiply in the host and in turn triggers the body’s immune response for long-term protection [34]. In another study, the authors [36] used overlap extension PCR to create a *Vibrio alginolyticus hopPmaJ* mutant strain with a 2600-fold reduction in virulence and the ability to generate various immune responses in orange-spotted grouper (*Epinephelus coioides*). The grouper vaccinated with *hopPmaJ* through intraperitoneal injection showed a high level of protection against virulent *V. alginolyticus* challenge by producing a high antibody titer with a relative percent survival (RPS) value of 84% [36].

Vaccination is a safer and more effective way than antibiotics to prevent and control vibriosis in aquaculture. Vaccination not only reduces the use of antibiotics, but it can also trigger a powerful immunological response [37]. However, inactivated vaccines have the disadvantages of a large inoculation dose, a short immunization time, and a single immunization route, which requires multiple immunizations. Inactivated vaccines mostly boost humoral immunity while failing to boost mucosal immunity [34]. It was discovered that formalin-killed cells of *Edwardsiella tarda* vaccination failed to protect ginbuna crucian carp against *E. tarda* infection [38]. Live attenuated vaccines are associated with risk of infection in people with weakened immune systems, and mutations may increase virulence [34]. There are various issues with vaccination, such as difficult administration, the need for a large labor force, and higher costs due to multiple administration to maintain the immunity of hosts. The vaccines against *Vibrio* spp. are mostly administered to fish via intraperitoneal injection. Their administration is only possible in fish of suitable body sizes and it needs skilled personnel to conduct the injection with minimum stress. Otherwise, the injection can be stressful for fish, making them more susceptible to infection [39]. Therefore, the development of *Vibrio* spp. immersion or oral vaccines could be a new strategy to control vibriosis. Despite the positive effects of vaccines, there is debate concerning the use of vaccinations in invertebrates. Theoretically, invertebrates cannot respond to vaccines in a selective manner because they lack the specific and adaptive immune system of vertebrates [40].

### 3.3. Probiotics

Probiotics are live microbial feed supplements that help to maintain the microbial balance in the host’s intestine [41]. Probiotics are microbial cells that are supplied through the gastrointestinal tract to improve the health of the fish [42]. A probiotic can also be described as a live microbial supplement that has a variety of beneficial impacts on the host, such as changing the host’s ambient microbial population, improving the nutritional content of feed ingredients, and improving the host’s immune responses to pathogens [43]. These microbial compounds can be delivered to fish in the form of feed or water supplements to improve disease resistance, health, growth performance, and stress response modulation. Yeast, microalgae, Gram-positive bacteria, and Gram-negative bacteria can all be considered as probiotics [44]. *Bacillus*, *Lactococcus*, *Lactobacillus*, *Pseudomonas*, *Enterococcus*, *Aeromonas*, *Alteromonas*, *Bifidobacterium*, *Clostridium*, *Phaeobacter*, *Pseudoalteromonas*, *Rhodosporidium*, *Roseobacter*, *Streptomyces*, and several other bacterial species have been shown to be effective in aquaculture feed with unique and beneficial properties. *Bacillus* spp., among the most widely utilized probiotics, are also widely employed in aquaculture for moderating toxicity symptoms [45], and increasing immunity and antioxidant capacity [46].

For 60 days, Nile tilapias (*Oreochromis niloticus*) were fed with *Lactobacillus plantarum* 1KMT as a supplement food. As a result, the fish’s immunity, intestinal microbiota, survival rate, growth performance and resistance to the *V. parahaemolyticus* challenge were greatly improved by *L. plantarum* 1KMT [47]. The effects of a probiotic product containing *Bacillus subtilis*, *Bacillus licheniformis*, *Lactobacillus* spp., and *Arthrobacter* spp. on cobia (*Rachycentron canadum*) growth, non-specific immunity, and protection against *V. harveyi* infection were studied. For 8 weeks, the fish were fed with diets with various doses of probiotics. The study revealed that specific growth rate (SGR), serum lysozyme, alternative complement pathway (ACP) activity, phagocytosis percentage (PP), and respiratory burst activity of cobia head kidney macrophages were all significantly increased by dietary probiotics. Furthermore, fish that were fed with probiotics had a much lower mortality rate when confronted with *V. harveyi* [48].

Although probiotics have many advantages, they also have some limitations. The most significant disadvantage to using probiotics is that they are often unable to maintain themselves and require supplementation regularly, making this strategy less cost-effective. Moreover, the specificity and longevity of probiotics for protection is uncertain [49]. Estimating an exact concentration to be administered is difficult and more study is needed to address this problem. Probiotic overdoses or extended treatments suppress the host’s ongoing immune responses [10]. However, if there are issues with the choice and usage of probiotics, they may have negative consequences and allow harmful species to spread and become more resistant to the host. The selection of probiotics is important, so it is essential to analyze their molecular mechanisms prior to use [50].

### 3.4. Phytotherapy

The use of available treatments is widely acknowledged to be linked to adverse effects. As a result, phytotherapy has continued to be a key area of research in the search for a safer, more precise, and more reliable treatment. The aquaculture industry favors phytotherapy since it is affordable and environmentally friendly [51]. Previous studies have proven that medicinal plants have strong antibacterial activity against *Vibrio* sp. It is known that the antimicrobial activity of plant extracts is correlated with their active compounds that can be found in various parts of the plant, such as the roots, leaves, fruits, seeds, and skin [52].

Karim et al. [53] reported that the extracts of *Desmodium triflorum* (L.) whole plant (5 mg/mL) and *Terminalia citrina (Roxb.)* (2.5 mg/mL) have antibacterial activity against *V. cholerae*. Both plant extracts exhibited zones of inhibition comparable to the commercially available meropenem antibiotic (10 μg/disk). The presence of tannins and alkaloids in the extracts may contribute to their antibacterial effects. In another study, *Psidium guajava* leaf, *Piper betle L.* leaf, *Phyllanthus amarus* leaf, *Rhodomyrtus tomentosa* seed, and *Allium sativum* bulb extracts were tested against *V. parahaemolyticus* and *V. harveyi* using the disk diffusion method. The findings revealed that *P. amanus* and *R. tomentosa* demonstrated the strongest antibacterial activities, followed by *P. guajava* and *P. betle*, whereas *A. sativum* had no bactericidal effects. For safety evaluation, white leg shrimps were fed with extract-coated feed pellets, and the survival rates were recorded. Only two extracts, *R. tomentosa* and *A. sativum*, were found to be safe for shrimps, whereas the others significantly reduced survival rates [54].

Various medicinal plant parts have been shown to have antibacterial effects; however, most studies on the potential benefits of these substances have focused only on in vitro experiments. Bioactive natural compounds are essential components of medicinal plant extracts, but their mechanisms of action remain unclear. Modern treatments from these extracts can only be created after thorough examination of their therapeutic properties, toxicity, and bioactive modes of action, as well as subsequent proper standardization in clinical trials [55]. The growing commercial demands for wild-sourced plant medicines bring about deforestation and degradation of species-rich forest ecosystems, presenting an additional challenge to utilizing plants as treatments. Some medical plants are prone to be endangered or threatened due to resource destruction [56].

## 4. Nanoparticles

Nanotechnology is one of the fastest-growing innovations in science and technology in recent years, and it has resulted in enormous development. Nanoparticles are tiny materials with a range of 1 to 100 nm in size, which exhibit a large surface-area-to-volume ratio [57]. The large surface area of nanoparticles makes them suitable for various applications, such as biomedical, drug delivery, optical, and electronic. Nano-sized particles have unique physical and chemical properties and are becoming increasingly important materials in the creation of new nanodevices for a variety of biological applications [58]. The attraction of these nanoparticles for biological applications arises from their important and distinct characteristics, such as their substantially higher surface-to-mass ratio than other particles, their quantum properties, and their ability to absorb and transport other compounds. Nanoparticles have a huge surface area that allows them to bind, absorb, and transport other molecules like medicines, probes, and proteins [59].

### 4.1. Nanoparticles in Aquaculture

Aquaculture is the fastest-growing primary sector, helping to meet the need for animal proteins and lipids. Aquaculture and fisheries provide around 15% of the average animal protein consumption of the world’s 2.9 billion people and are still growing. Aquaculture can provide employment and earnings in a variety of locations across the world [60]. However, contamination of the environment and the incidence of disease are seen as critical issues for the industry. In this regard, new technological methods have been introduced to cope successfully with such issues. Nanoparticles, as a fresh and innovative technique, have a wide range of applications and huge potential in aquaculture. Nanoparticles have recently been discovered to have potential in the prevention of microbial growth. Treatment with nanoparticles has been shown to efficiently reduce most bacterial infections, with the risk of bacterial resistance being minimal [61]. Antimicrobial properties are one of the most common uses for nanoparticles. Although nanoparticles were found to inhibit *Vibrio* spp. effectively, their toxicity to aquatic organisms must be evaluated. The study of nanoparticles in aquatic organisms is still lacking. Extensive research will be needed into the risk of accumulation in animals as well as the economic sustainability of large-scale production and application.

#### 4.1.1. Silver Nanoparticles

Silver nanoparticles (AgNPs) have risen in popularity as one of the most successful nanoparticles in a variety of sectors, displaying the greatest achievement as antibacterial agents. Li et al. [62] found that AgNPs have higher toxicity to microorganisms than eukaryotic cells or mammalian cells. Baskaralingam et al. [63] examined the inhibitory efficacy of green synthesized AgNPs from *Calotropis gigantea* leaf extract against pathogenic *V. alginolyticus*. In this work, the number of colonies of *V. alginolyticus* was successfully reduced by increasing AgNP concentrations. At 5 µg/mL of AgNP concentration, the number of colonies decreased, and at 20 µg/mL of AgNP concentration, *V. alginolyticus* was completely inhibited. Treatment with AgNPs effectively controlled *V. alginolyticus* in brine shrimp (*Artemia franciscana*). The authors revealed that infected *Artemia* cultures treated with AgNPs (10 µg/mL) had a higher survival rate (>40%) than those not treated with AgNPs. In another study, the antibacterial properties of AgNPs of two different sizes (16.62 and 22.22 nm) were tested against *V. harveyi*. The obtained results showed that smaller AgNPs had greater antibacterial activity against *V. harveyi* than larger nanoparticles. According to the findings of the study, AgNPs have potential as antibacterial agents for reducing bacteria growth [64]. The study by Huq [65] found that AgNPs produced using *Lysinibacillus xylanilyticus* strain MAHUQ-40 could be used as an antibacterial agent. *Vibrio parahaemolyticus* and *Salmonella Typhimurium* were reported to be inhibited by AgNPs with minimum inhibitory concentrations (MICs) of 3.12 and 12.5 µg/mL, respectively. Field emission scanning electron microscopy (FETEM) examination revealed that the AgNPs caused structural and morphological alterations, as well as disrupting the membrane integrity of bacteria. The nanoparticles’ positive surface charge electrostatically bound to the cell membrane’s negative charge, promoting the membrane adhesion of the nanoparticles. An obvious morphological change occurred as a result of the interaction, which was further characterized by cytoplasmic shrinkage and cell membrane disruption [66].

In another study, it was found that the generation of reactive oxygen species (ROS) may play a role in AgNP-induced cytotoxicity in *E. coli* and *S. aureus*. The formation of ROS by nanoparticles triggers a series of pathogenic processes, including inflammation, fibrosis, genotoxicity, and carcinogenesis [67]. The ROS production destabilizes plasma membrane integrity and lowers intracellular ATP levels, causing damage to the cellular respiratory chain (cellular enzymes) and DNA damage, which leads to cell lysis and death [68]. According to Dong et al. [69], the nanoparticles penetrated the bacterial cells, as shown by a transmission electron microscope (TEM) analysis. The small sized nanoparticles allow greater contact with the bacterial cell, as well as superior penetration [70]. Treatment with AgNPs causes alteration of the membrane structure, resulting in a considerable increase in permeability. This impairs the ability of bacterial cells to regulate transport across the membrane, leaving them unable to appropriately regulate transport across the plasma membrane, resulting in cell death [71].

#### 4.1.2. Gold Nanoparticles

Among the most stable and promising metal particles are gold nanoparticles (AuNP). Due to their great biocompatibility and benign nature, AuNPs have recently attracted a lot of attention [72]. The protective potential of orally administered AuNPs against *V. parahaemolyticus* in shrimp was investigated. AuNPs were given to shrimp in single doses of 0.2, 2, and 20 µg/g feed. The AuNPs at 2 µg/g successfully reduced the histopathological damage and enhanced survival (80%) in shrimp challenged with *V. parahaemolyticus*. The administration of AuNPs in shrimp did not produce any symptoms of death or toxicity [73]. Babu et al. [74] reported that the green synthesis of AuNPs from marine red alga *Acanthophora spicifera* (As-AuNPs) showed antibacterial activity against *V. harveyi* and *S. aureus*. The biosynthesized As-AuNPs at various concentrations of 25, 50, 75, and 100 μg/mL were more effective against *V. harveyi* than against *S. aureus* in terms of antibacterial activity. Bacteria treated with the highest concentration of As-AuNPs (100 µg/mL) showed a significant increase in protein leakage activity [74]. In another study by Vijayakumar et al. [75], marine polysaccharide fucoidan from *Fucus vesiculosus* was used to synthesize gold nanoparticles (Fu-AuNPs) and the antibacterial efficacy was further evaluated against *Aeromonas hydrophila* in tilapia (*Oreochromis mossambicus*). At 100 µg/mL, the synthesised Fu-AuNPs displayed a significantly better antibacterial effect against *A. hydrophila* compared with the commercial antibiotic chloramphenicol. The mortality rate of infected fish that received treatment with Fu-AuNPs was lower (30%) compared with untreated infected fish (90%).

AuNPs were able to enhance antibacterial effects when combined with antibiotics. It was reported that cefotaxime-loaded gold nanoparticles (C-AuNPs) had stronger antibacterial properties than free cefotaxime and AuNPs. This was attributed to the fact that the AuNPs included a considerable amount of cefotaxime, which was easily absorbed by bacteria and was not subjected to extensive degradation by bacterial enzymes. The antibacterial effect of AuNPs may have resulted from their ability to damage the bacterial DNA, most likely by direct contact and by preventing unwinding during transcription [76]. The release of gold ions from AuNPs contributes to their antibacterial properties. The higher concentration of gold nanoparticles will release more gold ions (Au^+^) with a stronger antibacterial effect. The released Au^+^ is uniformly dispersed all over the bacteria, and penetrates the cell walls to gain entry into the bacteria. These ions are able to interact with thiol groups to create Au–thiol groups, which trigger protein coagulation that leads to death of the bacteria [77].

#### 4.1.3. Other Types of Nanoparticles

Among the gold, silver, palladium, tungsten, and other transition metal nanoparticles under research, copper nanoparticles are the least expensive [78]. In a study by Ghuglot et al. [79], copper nanoparticles (CuNPs) were synthesized using *Trigonella foenum-graecum* leaf extract, and *V. harveyi*, *V. parahaemolyticus*, and *V. vulnificus* were used to investigate the efficacy of biosynthesized CuNPs as an antibacterial agent. The zone of inhibition changes in a linear relationship with the concentration of CuNP (0.5 mM, 1 mM, 1.5 mM, 2 mM, and 2.5 mM). CuNP with an antibacterial action against *Vibrio* pathogens could be a suitable candidate treatment for vibriosis in aquaculture [79]. In studies in vitro and in vivo, Chari et al. [80] investigated the effects of CuNPs on *Vibrio alginolyticus*, *Vibrio parahaemolyticus*, and *Aeromonas hydrophila*. CuNPs at a very low concentration (100 ng/mL) showed antibiofilm action of >60%. In the in vivo study, CuNPs significantly increased the survival rate (>80%) of *Artemia salina* against *A. hydrophila*, *V. alginolyticus*, and *V. parahaemolyticus*, demonstrating the non-toxic action of CuNPs with a 100% survival rate. A possible mechanism for copper oxide nanoparticles’ mode of action against *E. coli* has been suggested by a study. Saidin et al. [81] reported that copper oxide nanoparticles are absorbed onto the cell surface, degrading the cell wall, and ultimately damaging the cell membrane, leading to increased cell membrane permeability and reducing bacterial viability in copper oxide solution.

Zinc oxide (ZnO) is a semiconductor with unique qualities including a strong excitation binding energy and a broad band gap, which has sparked a lot of interest and found use in multiple applications such as gas sensors, biosensors, photocatalysts, solar cells, and antibacterial agents [82]. ZnO is a nanomaterial that has been approved by the Food and Drug Administration (FDA) and is generally regarded as safe, effective, and non-toxic at low concentrations. Anand et al. [83] synthesized zinc oxide nanoparticles (ZnONPs) from *Halimeda opuntia* extract and tested their antibacterial efficacy against *V. harveyi*. The various doses of ZnONPs (1, 2.5, 5, 7.5, and 10 μg/mL) demonstrated different growth inhibition zones against *V. harveyi*. The antibacterial activity results showed that the ZnONP-treated concentrations inhibited *V. harveyi* growth in a dose-dependent and time-dependent manner. The antibacterial activity against *V. parahaemolyticus* of zinc oxide (ZnO), copper oxide (CuO), and selenium (Se) nanoparticles made from the marine brown alga *Sargassum swartzii* was investigated. The MIC of ZnO, CuO, and Se nanoparticles was 25, 25, and 10 μg/mL, respectively. ZnONPs were effective as an antibacterial agent due to the production of ROS, which caused severe bacterial damage [84]. The production of ROS (OH^−^, H_2_O_2_, and O_2_^2−^) was observed [85]. The superoxide and hydroxyl radicals were unable to penetrate the membrane due to their negative charges. Therefore, these species were present on the bacteria’s outer surface. However, H_2_O_2_ molecules can penetrate through the bacterial cell wall [86]. Organelle membranes can rupture due to excessive ROS, allowing the organelles’ contents to leak [87]. As a result, damage and destruction occur, eventually leading to cell death [88].

Although nanoparticles have been used for various applications, some issues arise in the use of nanoparticles. The application of nanoparticles is limited due to their toxicity, stability, and safety. It was claimed that ZnONPs were not stable in aqueous solution. In a study by Tso et al. [89], ZnONPs were broken down into nano-meter sizes by an ultrasonic disruptor. After 2 h, ZnO particles were shown to aggregate abruptly, producing micro-meter particles that were larger than 1 μm [89]. ZnO particles are unable to maintain their particle size due to ZnO particles being converted into zinc ions, making them unable to stabilize electrostatically ZnO suspensions [90]. A high concentration (123.7 and 265.1 μg Ag/g) of Ag was found in shrimp (*Penaeus duorarum*) hepatopancreas after exposure to 1 and 10 μg Ag/L. The accumulation of Ag in the hepatopancreas was dose-dependent, indicating a crucial role for this organ in silver detoxification and accumulation [91]. Different sizes of AuNPs were detected in different organs of Wistar rats. The intestine accumulated the majority of 10 nm AuNPs, while the spleen tended to accumulate 30 nm and 60 nm AuNPs. The study reported that nanoparticles are not expelled in urine because the size of particles exceeds the renal filtration cutoff; instead, they are removed from the circulation through the reticuloendothelial system, which causes them to accumulate in the spleen [92]. The antibacterial effects of various nanoparticles are summarized in Table 1.

Concerns over the impact of nanoparticles on human health and the environment have increased. Workers in the nanotechnology industry are more likely to be exposed to nanoparticles during the production, transportation, and application of materials. As nanoparticle applications thrive in myriad industries, there are higher risks of skin exposure to nanoparticles, inhalation, and ingestion, which could lead to these nanoparticles reaching the bloodstream, with unknown adverse effects on vital organs [93]. Human skin is the main defense against foreign substances; however, the small size of nanoparticles is able to penetrate the barrier through hair follicles and sweat glands [94]. According to Larese et al. [95], electron microscopy was able to detect AgNPs (25 nm) on both intact and damaged skin. The study discovered wider silver skin penetration in damaged skin compared with intact skin. Additionally, inappropriate handling of industrial waste can have negative effects on the aquatic and soil environment. Green algae *Chlorella vulgaris* has been used to test the toxicity of superparamagnetic iron oxide nanoparticles (SPIONs). It is known that algae are indicators of the health of aquatic ecosystems due to their capacity to bioaccumulate metallic pollutants. SPIONs caused significant toxicity, inhibited the photochemical activity of algal cells by generating oxidative stress, and inhibited cell division [96]. Ploeg et al. [97] revealed that the exposure of earthworms, *Lumbricus rubellus*, to AgNPs resulted in a reduction in growth and production rate. The analysis of treated soil samples showed that single AgNPs and AgNP clusters were detected in the soil.

The application of graphene oxide (GO) in aquaculture is considered a new aspect for researchers to explore. GO has shown antibacterial activity against Gram-negative and Gram-positive bacteria, but the study of its effects against aquaculture bacteria is still limited.

## 5. Graphene Oxide (GO)

**Figure 2:**
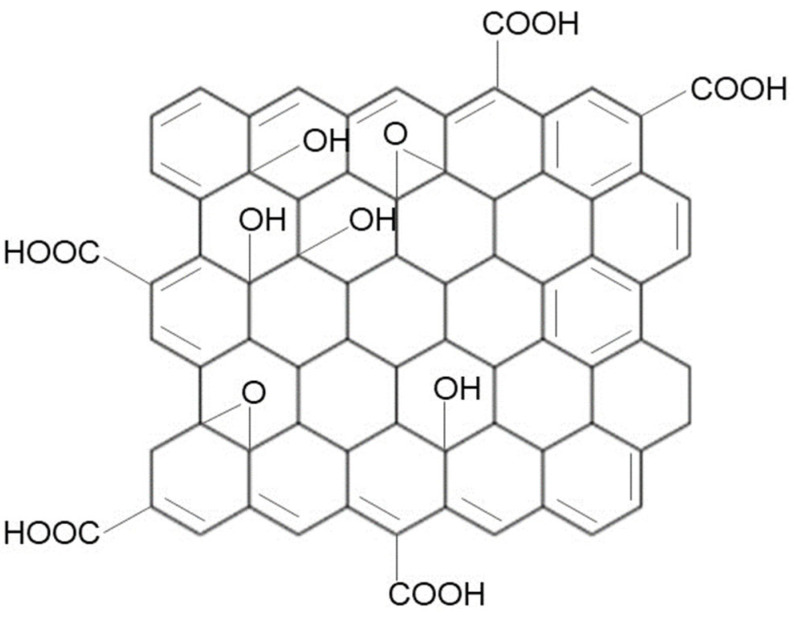
Graphene oxide structure. GO is an oxidized form of graphene that consists of phenyl epoxide (-O-), hydroxyl (-OH), and carboxylic acid (-COOH) groups.

Among graphene-based materials, graphene oxide (GO) is considered the most well-known graphene derivative. GO has received a lot of attention in recent years due to its characteristic two-dimensional (2D) and single-atom-thick structure that is relevant to biological applications. GO is a highly oxidized form of graphene that has been exposed to a variety of strong oxidizing chemicals [98]. GO has a layered structure similar to graphite, but the carbon atoms’ planes are extensively decorated by oxygen-containing groups in GO. Figure 2 shows that GO is made up of a graphene sheet with phenyl epoxide (-O-) and hydroxyl (-OH) groups on the basal plane and carboxylic acid (-COOH) groups on the edges. The ionization of the carboxylic acid group leads to electrostatic repulsion, causing the monolayer to form an aqueous colloidal dispersion [99,100]. GO has two main characteristics: (1) it is highly hydrophilic and can form stable aqueous colloids to facilitate the assembly of macroscopic structures using simple and economical solution processes; and (2) it can be produced using inexpensive graphene as the basic material and by using reasonable chemical methods with a high yield [101].

The bactericidal activity of GO was previously tested against *E. coli*, showing time- and concentration-dependent behaviors. The majority of the bacteria was inactivated during the first hour, and the rate of cell death went up in correlation with the concentration of GO [102]. The same author also revealed that the antibacterial action of GO sheets against *E. coli* cells is influenced by their lateral size, finding that bigger GO sheets had stronger antibacterial activity than smaller GO sheets and demonstrated different concentration-dependent and time-dependent behaviors. After 1 h of incubation with large GO sheets, the majority of *E. coli* cells were damaged, whereas after 4 h of incubation with small GO sheets, the inactivation rate of *E. coli* cells continued to rise [103]. Using the plate-counting methodology, the antibacterial activity of GO and GO sheets decorated with silver nanoparticle (GO–Ag) nanocomposite against *P. aeruginosa* was examined. Over the concentration range studied (0.1–5.0 μg/mL), GO showed no antibacterial activity, whereas the GO–Ag nanocomposite had a strong antibacterial effect with a minimum inhibitory concentration ranging from 2.5–5.0 μg/mL [104]. GO was investigated for its antibacterial activity against *Xanthomonas oryzae pv. oryzae* (*Xoo*). The findings demonstrated that, even at low concentrations (250 µg/mL), GO had exceptional bactericidal activity, killing 94.48% of cells [105]. In another study by Gao et al., the antibacterial effect of GO was tested against both Gram-negative bacteria *E. coli* and Gram-positive bacteria, *S. aureus*. According to the results, GO worked well on both Gram-positive and Gram-negative bacteria, with *S. aureus* being more affected than *E. coli* [106]. Wu et al. [107] studied the antibacterial effect of GO against multidrug-resistant (MDR) bacteria, Klebsiella pneumoniae (*K. pneumoniae*). The survival rate of *K. pneumoniae* dropped as low as 3.2% at the highest GO concentration (500 μg/mL). Research conducted in our laboratory has also revealed the potential anti-bacterial effects of GO when incorporated with gallic acid (GAGO) to inhibit methicillin-resistant *S. aureus* (MRSA). The ability of GAGO to inhibit the growth of MRSA was observed at ≥150 µg/mL. The findings suggest that GAGO could be developed as an alternative antibacterial agent against multidrug-resistant bacteria [108,109].

**Figure 3:**
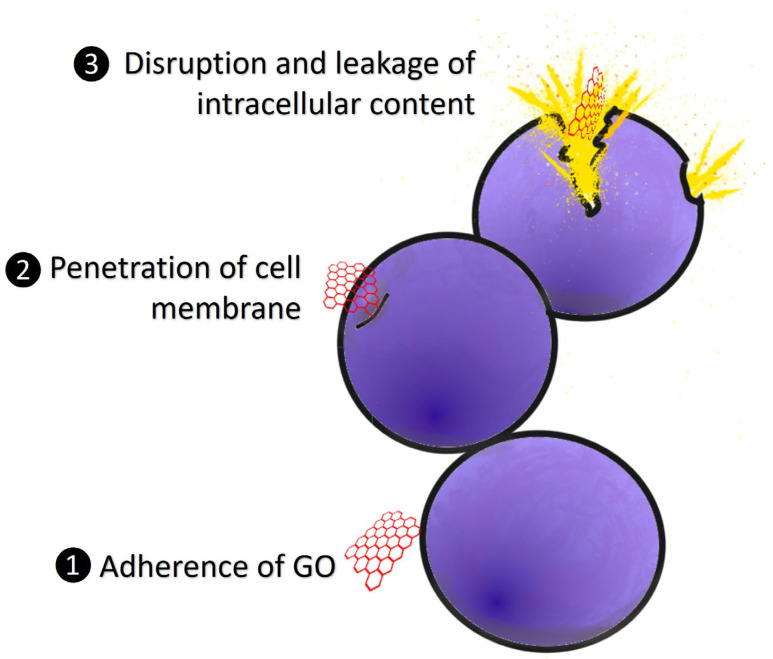
The antibacterial mode of action for GO. (1) GO adheres to the bacterial membranes. (2) The sharp edges of GO penetrate the cell membranes. (3) The damaged cell membrane leads to disruption and leakage of intracellular content.

The antibacterial activities of GO were mostly due to the physical and chemical interactions between GO and bacterial cells [110]. Figure 3 displays a summary of the antibacterial modes of action for GO. The bacterial cell membrane has been identified as a significant target for GO cytotoxicity studies [111]. Changes in the transmembrane potential, morphological changes in the cell structure, leakage of RNA and internal electrolytes, and uptake of membrane-impermeable dyes have all been used to indicate membrane damage in GO-exposed bacteria [109,112,113]. The sharp edges of GO enable it to penetrate the cell membrane, and the disruption of membrane integrity is the primary factor for membrane damage [103]. The sharp edges of the graphene nanosheets cut through the bacterium’s cell membrane, allowing the intracellular matrix to leak and leading to death [114]. According to Akhavan et al. [115], direct contact of the sharp edges of GO with bacteria triggered RNA effluxes through the damaged cell membranes of *E. coli* and *S. aureus*. An obvious morphological change occurred as a result of the interaction, which was characterized by cytoplasm shrinkage and cell membrane disruption. According to a few studies, GO sharp edges may not be responsible for its antibacterial properties alone, but adsorption on the GO basal planes plays a part in bacterial membrane damage [116,117]. Nonetheless, the main mechanism of GO’s cytotoxic effects on bacteria is membrane disruption.

### 5.1. Application of Graphene Oxide in Aquaculture

Early detection and routine diagnosis of diseases in aquaculture has warranted various research with a view to finding a reliable, simple, an effective method for rapid screening. Sarkar et al. [118] described nanosensors as useful to act as simple tools for detecting aquaculture pathogens. GO is currently being used as a template in the development of electrochemical biosensors due to its unique chemical composition and biocompatibility. A simple and selective GO-based electrochemical immunosensor for the rapid detection of white spot syndrome virus (WSSV) in raw infected shrimp tissue samples was discovered for the first time. Unlike PCR amplification-based detection approaches, this is a unique and alternative qualitative and quantitative method [119]. Sha et al. [120] created a label-free electrochemiluminescence (ECL) immunosensor for detection of *V. parahaemolyticus* in seawater and sea food by using multi-functionalized GO (nanoFe_3_O_4_@GO). This approach was created by immobilizing N-(4-aminobutyl)-N-ethylisoluminol (ABEI) and *V. parahaemolyticus* antibodies on the surface of nanoFe_3_O_4_@GO. The immunosensor was effectively employed to evaluate the concentration of *V. parahaemolyticus* in seawater and sea food, demonstrating good detection capabilities, such as high sensitivity and selectivity, as well as good stability. The limit of detection for this approach was reported to be 5 CFU/mL. Interestingly, another study also utilized multi-functionalized GO, and the ECL immunosensor was found to detect *V. vulfinus* successfully. The developed immunosensor has a detection limit as low as 1 CFU/mL [121].

GO has also been tested against. *Aeromonas hydrophila* (*A. hydrophila*), *Edwardsiella tarda* (*E. tarda*), *Streptococcus* spp., and *Vibrio* spp., which are some of the well-known bacteria that threaten aquatic animal health. Wei et al. [122] revealed that GO exhibited antibacterial activity against *A. hydrophila*, *E. tarda*, *Flavobacterium* spp., *Pseudomonas* spp., and *Streptococcus* spp. This is further supported by a recent study by Lee et al. [123], which demonstrated the ability of GO to impose maximum anti-bacterial activity with 100% inhibition against *A. hydrophila*, *S. parauberis*, *S. iniae*, and *P. piscicola* following 24 h of exposure. In addition to this, GO has also been developed to serve as an absorbent for antibiotic residue in aquaculture. In a previous study, GO successfully removed tetracycline from aqueous solution. It is reported that aromatic compounds in tetracycline can be readily adsorbed on GO by π-π interaction [124].

There has been research into the use of GO in the detection of pathogens in aquaculture, but the use of GO in the management and mitigation of vibriosis is still unknown. Research on this aspect is warranted to explore the potential of GO as an alternative for the treatment of bacterial infections in aquaculture. This could be a good strategy to reduce the use of antibiotics in the aquaculture sector.

### 5.2. Antibacterial Effects of Graphene Oxide against Vibrio Spp.

The research on the antibacterial effects of GO against *Vibrio* spp. has been studied in previous reports. Wei et al. [122] tested the MIC of GO against bacteria isolated from aquaculture sites. In that work, the *Vibrio* spp. used were *V. harveyi*, *V. parahaemolyticus*, and *V. alginolyticus*, which were isolated from *Lates calcarifer* (Asian seabass), *Anadara granosa* (blood cockle), *Penaeus monodon* (black tiger shrimp), and *Litopenaeus vannamei* (white leg shrimp). After 24 h of incubation with GO at concentrations ranging from 0.244 to 500 mg/L, the growth of bacteria was analyzed. Inhibitory action against GO was observed in all *Vibrio* isolates at concentrations ranging from 7.81 to 125 mg/L. In another study, *V. harveyi* and *V. scophthalmi* cultures were incubated with GO polyester fiber for 1, 6, and 12 h to observe the colony-forming units (CFU). After the complete incubation period, *V. harveyi* was identical to the control group. In comparison to the control group, the number of *V. scophthalmi* was reduced, but there were no changes over the incubation time. As a result, GO did not exhibit any antibacterial activity against *V. harveyi* and *V. scophthalmi*. Due to the strong flagellar mobility of the genus *Vibrio*, which has peritrichous flagella, it was reported that the bacteria were not trapped in the fibers and were unaffected by GO [123].

### 5.3. Challenges and Future Applications of Graphene Oxide in Aquaculture

The concerns about the safety and toxicity of materials remain unanswered, and great care is being taken to analyze their toxicity. Many prior studies have shown that GO could be useful in the real world while exhibiting low cell toxicity, but the results vary. According to reports, graphene’s toxicity in biological systems is strongly influenced by its concentration, lateral size, surface structure, functional groups, purity, and protein corona [125]. All of these conditions can cause a wide range of biological reactions. To exploit GO in real-world applications, considerable in vitro and in vivo research using cells and animal models is required to demonstrate its toxicity. The effects of GO on aquatic organisms were tested on zebrafish (*Danio rerio*) either as embryos or adults due to their easy maintenance and close homology with the human genome [126].

The cytotoxicity, genotoxicity, and oxidative stress effects of GO on adult zebrafish were investigated. The number of gill cells in the early stages of apoptosis and necrosis increased after exposure to GO at concentrations of 2, 10, and 20 mg/L. In gill cells, the production of reactive oxygen species (ROS) was noticed. Gill tissue injuries including a dilated marginal channel, lamellar fusion, clubbed tips, swelling mucocytes, epithelial lifting, aneurysms, and necrosis were observed [127]. Gills are the organism’s first line of defense against pollutants in the aquatic environment, so their defenses are activated quickly in abnormal situations [128]. The toxicological effects of GO on zooplankton (*Daphnia magna*) were investigated, and the findings demonstrated non-severe acute toxicities of GO on *D. magna*, including immobility (EC_50_ = 44.3 mg/L) and mortality (LC_50_ = 45.4 mg/L) at 72 h. After the first 24 h of exposure, high GO concentrations (20 mg/L) and after 48 h, all tested GO concentrations (5–50 mg/L) caused a significant increase in superoxide dismutase (SOD) activity. Under the microscope, *D. magna* showed rapid absorption of GO at a concentration of 10 mg/L. After 12 h of exposure, GO had filled most of the gut and had completely filled the gut after 24 h. According to the findings, GO can accumulate in the gut of *D. magna* and cause severe oxidative damage [129]. On the other hand, the study discovered that GO has a minor harmful effect on *Artemia franciscana* (brine shrimp). At the highest dose (100 μg/mL) and 72 h of exposure, GO significantly increased adult mortality by 25%. However, in adult *A. franciscana*, GO did not affect ROS generation, cholinesterase activity, nor growth rate for 72 h [130].

Based on previous study [127,129,130], GO at high concentrations demonstrated chronic toxicity in an animal model. As a result, it is critical to investigate ways to reduce the toxicity of GO so that it can be used safely in the future. Surface coatings and size have important roles in influencing material characteristics such as biodistribution, excretion, and toxicity [131]. The in vivo toxicity of GO can be successfully reduced with suitable surface modification [132]. In a study by Ghafor et al. [126], GO was loaded with gallic acid (GA) and the toxicity effects on zebrafish embryos were determined. In comparison to gallic acid-loaded graphene oxide (GAGO), pure GA and GO were used. GAGO successfully lowered the toxicity of pure GO and GA in zebrafish embryos by improving survival, hatching, and heart rate. GAGO had a safe concentration range of 0–150 μg/mL, which was higher than pure GA and GO. GAGO was also shown to reduce the generation of ROS, which could be one of the reasons for mortality of embryos, hence mitigating the toxic effects of GO [126]. In another study, coating with Pluronic F127 (PF) on the surface of GO was shown to reduce the toxicity of GO during embryonic development at different concentrations of 0–100 μg/mL [133]. At higher concentrations and longer exposure times of GO, the toxicity assessments in terms of survival, heart, and hatching rates were significantly affected. The data demonstrated that the toxicity of GO could be mitigated by functionalizing the surface of GO with a surfactant such as PF, which mainly consists of ethylene oxide and propylene oxide monomers [133].

GO has been found to be able to inhibit the growth of various pathogens. Therefore, the use of GO could be an alternative to antibiotics in controlling and preventing bacterial infections. Despite several studies on its antibacterial effectiveness, its impacts on animals and the environment during practical application remain unknown. The release of GO into the environment could endanger a wide range of living species. Efforts are needed to clarify the effects of using GO in the long term, aiming to prevent bacteria from developing resistance to GO. Research into GO stability in various environments is critical because it may have an impact on GO application.

In the application of nanomaterials in aquaculture, biodegradability, agglomeration, and precipitation are all important issues to consider. A biodegradable carrier is critical for delivering bioactive ingredients for increased efficacy and bioavailability. It should improve overall aquaculture management procedures through enrichment, resulting in reduced ingredient disposal, and it should be quickly absorbed and destroyed [118]. Further investigations are needed to analyze the degradation of GO.

## 6. Conclusions

Aquaculture industries are constantly provoked with challenges related to vibriosis due to several variables, such as *Vibrio* heterogeneity and adaptive abilities, as well as its abundant presence in the marine environment. Antibiotics, vaccinations, probiotics, and phytotherapy that are now accessible have their own weaknesses. Therefore, the development of new effective therapeutic and preventive measures for vibriosis should be prioritized. Traditional aquaculture techniques offer an opportunity for nanotechnology research and development. Nanoparticles have been used in a variety of biological applications, including antibacterial agents, and there is a possibility of using nanoparticles to control disease in aquatic organisms. There could be a good potential for using nanoparticles as antibacterial drugs in the long term as the nanoparticles impose various simultaneous mechanisms of action against bacteria, making it difficult for bacteria to acquire resistance. As a carbon-based material, GO has been utilized in a wide range of applications due to its unique characteristics. GO has antibacterial properties that can inhibit a wide range of microorganisms. The application of GO in aquaculture is still under consideration due to its toxicity at higher concentrations and the lack of study on its effect on the aquatic environment. However, GO can be functionalized with a variety of nanomaterials, including metal ion/oxide nanoparticles, polymers, enzymes, and bioactive compounds, to improve antibacterial action and reduce its toxicity.

## Figures and Tables

**Table 1:** Antibacterial activity of nanoparticles.

Nanoparticles	Concentration	Bacteria	Findings	Reference
AgNPs from *Calotropis gigantea* leaf extract	5–20 μg/mL	*V. alginolyticus*	*V. alginolyticus* was completely inhibited at 20 μg/mL and infected brine shrimp treated with AgNPs had a higher survival rate than the non-treated group	[63]
AgNPs	-	*V. harveyi*	Smaller AgNPs (16.62 nm) had greater antibacterial activity compared to larger particles (22.22 nm)	[64]
AgNPs produced using *Lysinibacillus xylanilyticus* strain MAHUQ-40	1.56–100 μg/mL	*V. parahaemolyticus* and *Salmonella Typhimurium*	AgNPs exhibited a MIC of 3.12 and 6.25 μg/mL for *V. parahaemolyticus* and *S. Typhimurium*	[65]
AuNPs	0.2, 2, and 20 μg/g feed	*V. parahaemolyticus*	AuNPs enhanced survival rate of challenged shrimps and did not cause any histological damage or toxic effects	[73]
AuNPs from marine red alga *Acanthophora spicifera*	25–100 μg/mL	*V. harveyi* and *S. aureus*	AuNPs showed greater antibacterial effect against *V. harveyi* than against *S. aureus*	[74]
fucoidan coated AuNPs	100 μg/mL	*Aeromonas hydrophila*	AuNPs effectively inhibited *A. hydrophila* and reduced the mortality rate of infected tilapia	[75]
CuNPs from *Trigonella foenum-graecum* leaf extract	0.5–2.5 mM	*V. harveyi*, *V. parahaemolyticus*, and *V. vulnificus*	CuNPs showed bactericidal effect against the bacteria	[79]
CuNPs	100 ng/mL	*V. alginolyticus*, *V. parahaemolyticus*, and *A. hydrophila*	CuNPs exhibited antibiofilm action of>60% and increased the survival rate of brine shrimp	[80]
ZnONPs from *Halimeda opuntia* extract	1–10 μg/mL	*V. harveyi*	Growth inhibition by ZnO increased with exposure duration and dose	[83]

## Data Availability

Not applicable.
